# Effect of a Novel Quaternary Ammonium Methacrylate Polymer (QAMP) on Adhesion and Antibacterial Properties of Dental Adhesives

**DOI:** 10.3390/ijms15058998

**Published:** 2014-05-20

**Authors:** Yasmine M. Pupo, Paulo Vitor Farago, Jessica M. Nadal, Luzia C. Simão, Luís Antônio Esmerino, Osnara M. M. Gomes, João Carlos Gomes

**Affiliations:** 1 Postgraduate Program in Dentistry, Department of Dentistry, State University of Ponta Grossa, Paraná 84030-900, Brazil; E-Mails: yasminemendes@hotmail.com (Y.M.P.); osnaramgomes@uol.com.br (O.M.M.G.); gomesjoaocarlos@uol.com.br (J.C.G.); 2 Laboratory of Pharmaceutical Products, Postgraduate Program in Pharmaceutical Sciences, Department of Pharmaceutical Sciences, State University of Ponta Grossa, Paraná 84030-900, Brazil; E-Mail: jessicabem@hotmail.com; 3 Multi-user Laboratory, State University of Ponta Grossa, Paraná 84030-900, Brazil; E-Mail: luziachaves4@hotmail.com; 4 Laboratory of Clinical Microbiology, Department of Clinical and Toxicological Analysis, State University of Ponta Grossa, Paraná, 84030-900, Brazil; E-Mail: esmerino@uepg.br

**Keywords:** antibacterial agents, bond strength, dentin-bond agents, quaternary ammonium compounds, polymerization, raman spectroscopy

## Abstract

This study investigated the resin–dentin bond strength (μTBS), degree of conversion (DC), and antibacterial potential of an innovative adhesive system containing a quaternary ammonium methacrylate polymer (QAMP) using *in situ* and *in vitro* assays. Forty-two human third molars were flattened until the dentin was exposed and were randomly distributed into three groups of self-etching adhesive systems: Clearfil™ SE Bond containing 5% QAMP (experimental group), Clearfil™ Protect Bond (positive control) and Clearfil™ SE Bond (negative control). After light curing, three 1 mm-increments of composite resin were bonded to each dentin surface. A total of thirty of these bonded teeth (10 teeth per group) was sectioned to obtain stick-shaped specimens and tested under tensile stress immediately, and after 6 and 12 months of storage in distilled water. Twelve bonded teeth (4 teeth per group) were longitudinally sectioned in a mesio-to-distal direction to obtain resin-bonded dentin slabs. *In situ* DC was evaluated by micro-Raman spectroscopy. *In vitro* DC of thin films of each adhesive system was measured using Fourier transform infrared spectroscopy. *In vitro* susceptibility tests of these three adhesive systems were performed by the minimum inhibitory/minimum bactericidal concentration (MIC/MBC) assays against *Streptococcus mutans*, *Lactobacillus casei*, and *Actinomyces naeslundii*. No statistically significant difference in μTBS was observed between Clearfil™ SE Bond containing 5% QAMP and Clearfil™ SE Bond (*p* > 0.05) immediately, and after 6 and 12 months of water storage. However Clearfil™ Protect Bond showed a significant reduction of μTBS after 12 months of storage (*p* = 0.039). In addition, QAMP provided no significant change in DC after incorporating into Clearfil™ SE Bond (*p* > 0.05). Clearfil™ SE Bond containing 5% QAMP demonstrated MIC/MBC values similar to the positive control against *L. casei* and *A. naeslundii* and higher than the negative control for all evaluated bacterial strains. The use of QAMP in an adhesive system demonstrated effective bond strength, a suitable degree of conversion, and adequate antibacterial effects against oral bacteria, and may be useful as a new approach to provide long-lasting results for dental adhesives.

## 1. Introduction

The risk of recurrent caries can be increased by residual bacteria in tooth restoration, which remains a challenge in dentistry [1,2]. In order to avoid this adverse condition, adhesive systems should present a long-lasting antibacterial activity for bacteria that enter this interface, leading to microleakage [1–3]. Accordingly, some antimicrobials freely dispersed into adhesive systems such as antibiotics, quaternary ammonium compounds glutaraldehyde, and fluorides have been extensively evaluated [4–11]. However these freely dispersed agents are inappropriately released from restorative material and result in decreased bond strength for restoration [11,12].

Other studies assessed the use of quaternary ammonium-based antibacterial monomers into dental resins such as 1,2-methacryloyloxydodecylpyridinium bromide (MDPB) [13–21]. MDPB was first synthesized 20 years ago and demonstrated antibacterial activity against *Streptococcus mutans*, *Lactobacillus casei*, and *Actinomyces naeslundii* [21–24]. This monomer was incorporated into an adhesive system and is now commercially available as Clearfil™ Protect Bond [20].

Despite that the use of quaternary ammonium monomers into adhesive systems can be considered a feasible approach, some studies have reported limitations in their use. Feuerstein *et al.* [1] performed a study to investigate the immediate and long-lasting antibacterial properties of four self-etching adhesive systems, including Clearfil™ Protect Bond for 14 days. Clearfil™ Protect Bond exhibited an antibacterial effect for only seven days. Moreover, no adhesive system showed antibacterial effect against *S. mutans* in 14 days. Thus the MDPB molecules that were not polymerized were leached out of the adhesive system leading to a higher antibacterial effect but only in a reduced time interval. In addition, quaternary ammonium compounds of low molecular weight as MDPB can have cytotoxicity to human pulp cells, which compromises their clinical safety [24,25].

**Figure 1.**
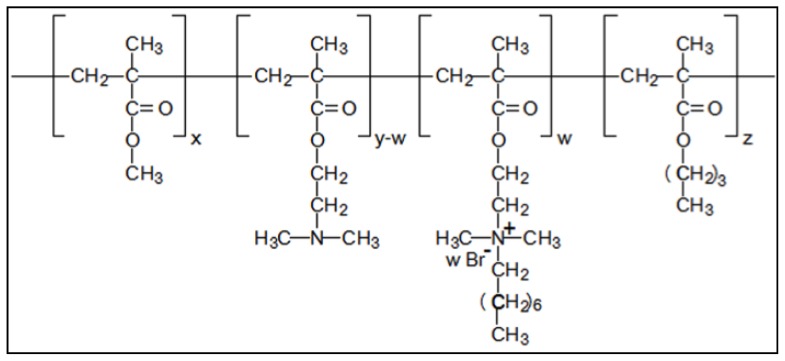
Chemical structure of the quaternary ammonium methacrylate polymer (QAMP) [Brazil patent application number 10 2012 0266 4] [28].

Novel antimicrobial agents are needed and much work has been devoted to developing highly efficient compounds that are also less susceptible to development of resistance by bacteria [26]. In a previous paper, our group successfully synthesized an innovative quaternary ammonium methacrylate polymer (QAMP), a poly(dimethylaminoethyl methacrylate-*co*-octyldimethyl ammonium ethyl methacrylate bromide*-co*-methyl methacrylate-*co*-butyl methacrylate) (Figure 1) [27]. Quaternary ammonium methacrylate polymer (QAMP) was properly characterized by Fourier transformed infrared spectroscopy, nuclear magnetic resonance spectroscopy, wide-angle X-ray powder diffraction, and thermogravimetric analysis and was then incorporated into a self-etching adhesive system [27]. The self-etching adhesive containing QAMP had antibacterial activity against *S. mutans* and was particularly recommended for reducing recurrent caries. Due to its high molecular weight, QAMP demonstrated a lower migration from the adhesive system compared to MDPB [27]. In addition, QAMP is a non-volatile and chemically stable polymer that is not permeable through the oral mucosa in contrast to the main problems of the chemically-related quaternized monomers [25]. However, further studies are required to evaluate the behavior of QAMP in adhesive systems concerning their physicochemical properties, e.g., resin–dentin bond strength (μTBS) and degree of conversion (DC) which are strongly related to dental restoration longevity. In addition, its antibacterial potential against other oral bacteria that compromise the durability of composite resin restorations remains unknown.

The microtensile test is a laboratory procedure used to quantify the bond strength of adhesive biomaterials to tooth substrates [29]. This assay shows a pre-clinical value for dental adhesives, based on the assumption that the stronger the tooth-biomaterial adhesion, the more effectively it will resist the stress imposed by polymerization shrinkage of the composite resin [30]. The composite resin generates contraction stresses that affect the cavity margin. In clinical situations, these stresses are responsible for the composite pulling away from the margin and create a marginal gap [31]. This detachment of the composite resin from the dental hard tissues can result in postoperative sensitivity, enamel cracking, recurrent caries, marginal discoloration and finally failure of the restoration [31,32]. The microtensile test can also provide insights about the strength of adhesion of restorative materials used in dentistry as a function of time [33].

The degree of conversion plays a crucial role in the long-lasting resin/dentin bond study due to monomer conversion after polymerization of light-cured materials, which can affect various mechanical properties of the adhesive system including the tensile, compressive, and flexural strengths, elastic modulus, wear, and hardness. High DC can also reduce permeability at the bond interface, which increases resistance to degradation [34]. Moreover an incomplete polymerization of adhesive monomers has been reported as one of the reasons for nanoleakages [35].

Antibacterial evaluation plays an important role during the development of newer restorative materials in order to provide additional properties for preventing and treating caries lesions [16]. The ability to control bacteria is required to eliminate the risk of further demineralization and cavitation, since dental caries is an infectious disease and its eradication is crucial for improving the durability of restorations [16]. Regarding the *in vitro* susceptibility tests, agar-disc diffusion is a traditional method that can be used. However its results do not distinguish whether the restorative materials exhibit bactericidal or bacteriostatic effects; further, the production of inhibition zones on the agar plate indicates only that bacterial growth was hindered [20]. In that sense, minimum inhibitory/bactericidal concentrations (MIC/MBC) are currently more recommended due to their quantification of intrinsic antibacterial activity that provides more suitable data for evaluating eradication of pathogenic oral bacteria, prevention of caries lesions and longevity of dental restorations [20].

Considering these factors, the objective of this paper was to characterize the QAMP by micro-Raman spectroscopy and to investigate μTBS and DC of a self-etching adhesive system containing QAMP in order to explore whether this quaternary ammonium methacrylate polymer can affect the dental restoration longevity. The null-hypothesis tested was that there is no difference in bond characteristics and degree of conversion among the primer/bond-resin containing QAMP and other control groups. Furthermore, this paper also aimed at assessing the *in vitro* antibacterial effect of a commercial adhesive system containing QAMP against the usual pathogenic oral bacteria for evaluating its potential on preventing caries lesions.

## 2. Results

### 2.1. Micro-Raman Spectroscopy of Quaternary Ammonium Methacrylate Polymer (QAMP)

**Figure 2.**
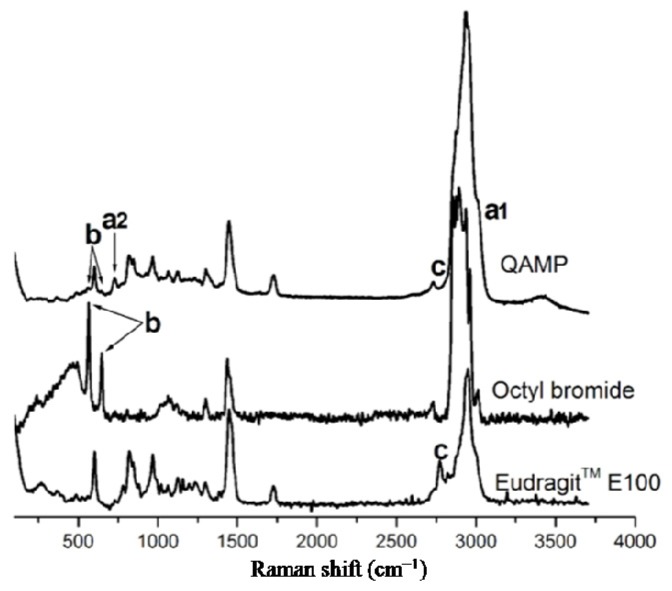
Raman spectra of QAMP and raw materials (Eudragit™ E100 and *n-*octyl bromide). (a1) Raman band at 3030 cm^−1^ as a broadening of C–H alkyl stretching bands; (a2) A novel band attributed to the symmetric stretching vibration of octyl dimethyl ammonium substituent was verified at 726 cm^−1^; (b) The C–Br stretching vibration presented in *n*-octyl bromide between 560 and 650 cm^−1^ disappeared in QAMP; and (c) Raman bands associated with dimethylamino groups of Eudragit™ E100, previously observed at 2772 and 2821 cm^−1^ also disappeared.

Raman spectra of the QAMP and raw materials (Eudragit™ E100 and *n-*octyl bromide) are presented in Figure 2. Eudragit™ E100 showed a C–N stretching band at 602 cm^−1^. An assignment at 818 cm^−1^ corresponded to C–C stretching vibration [36]. A Raman band at 1450 cm^−1^ was attributed mainly to –CH_2_– scissoring modes. A typical band at 1728 cm^−1^ was assigned to C=O ester stretching. Dimethylamino groups led to bands at 2772 and 2821 cm^−1^. A strong band due to C–H stretching was observed at 2944 cm^−1^. The Raman spectrum of this polymer also displayed bands at 1192, 1123, and 967 cm^−1^, which were associated with C–H and C–C wagging vibration [37]. Concerning *n-*octyl bromide, its Raman spectrum showed a typical band corresponding to C–Br stretching between 560 and 650 cm^−1^. In addition, vibrations of –CH_2_– and –CH_3_ groups were assigned at 1440 cm^−1^. Strong bands due to C–H stretching were observed between 2850 and 3000 cm^−1^.

Considering the spectrum of QAMP, Raman bands appeared at 3030 cm^−1^ (a1) as a broadening of C–H alkyl stretching bands. A novel band attributed to the symmetric stretching vibration of octyl dimethyl ammonium substituent was verified at 726 cm^−1^ (a2). These Raman bands were particularly related to the formation of the quaternary ammonium methacrylate polymer since these assignments were not previously observed in Eudragit™ E100 and *n-*octyl bromide. Similar Raman shifts were reported by Pigorsch *et al.* [38] in a study evaluating the spectroscopic characterization of cationic quaternary ammonium starches. As expected, the C–Br stretching vibration presented in *n*-octyl bromide between 560 and 650 cm^−1^ (b) disappeared in QAMP. Moreover Raman bands associated with dimethylamino groups of Eudragit™ E100, previously observed at 2772 and 2821 cm^−1^ (c), also disappeared. These Raman bands support an experimental basis for affirming that the quaternary ammonium methacrylate polymer was successfully synthesized. These data also reinforce the spectroscopic characterization previously performed by FTIR and described in our previous study [30].

### 2.2. Resin–Dentin Bond Strength (μTBS)

The results of μTBS (MPa) are summarized in Table 1. The mean cross-sectional area ranged from 0.80 to 0.95 mm^2^ and no difference among groups was verified (*p* > 0.05). Two-way ANOVA revealed that the interaction adhesive system *vs.* storage period was statistically significant (*p* = 0.02). A significant decrease on μTBS value was achieved for Clearfil™ Protect Bond after 12 M in water storage (*p* = 0.039). However, QAMP provided no statistical changes in μTBS after its incorporation into Clearfil™ SE Bond when compared to the negative control (*p* > 0.05). The percentage of specimens with premature debonding and the frequency of each fracture mode are shown in Table 2. The major fracture mode was adhesive/mixed for the three adhesive systems under study immediately, and after 6 and 12 months of storage in distilled water.

### 2.3. In Situ Analysis of Degree of Conversion (DC)

**Figure 3.**
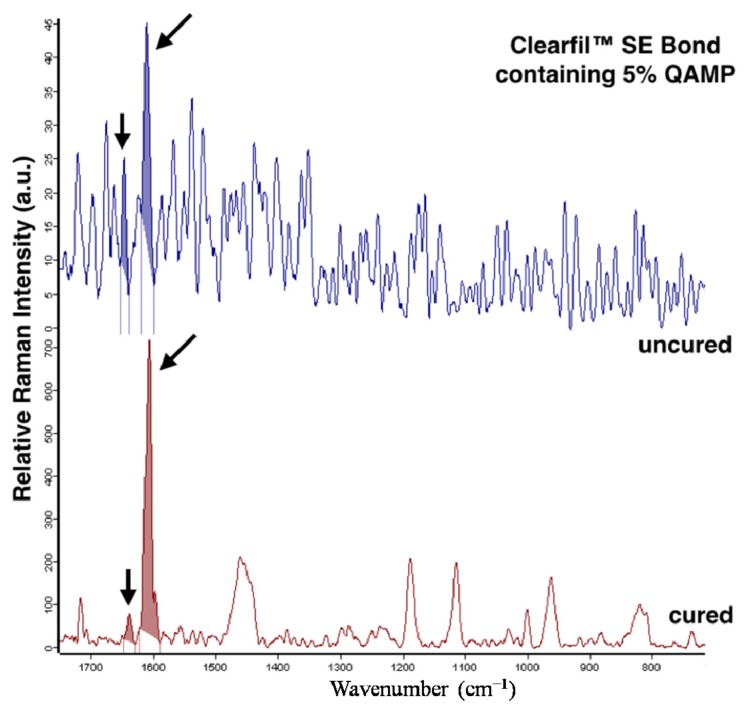

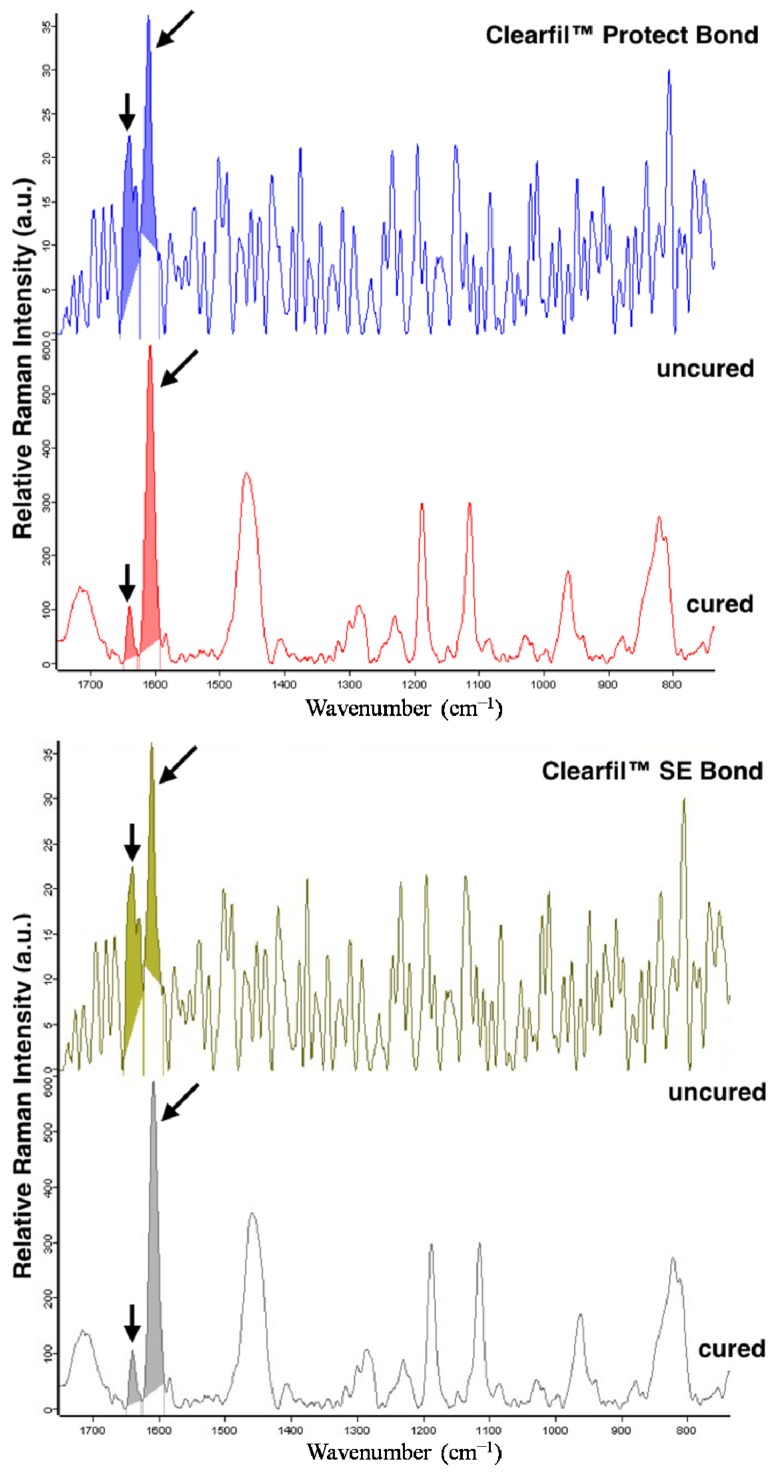
Micro-Raman spectras of uncured and cured adhesive systems Clearfil™ SE Bond containing 5% QAMP, Clearfil™ Protect Bond, Clearfil™ SE Bond. The reference band is shown at 1609 cm^−1^ and the reactive band related to C=C group is indicated at 1639 cm^−1^ (arrows). This band decreases during polymerization and was used for calculating the degree of conversion.

Figure 3 shows representative spectra of the evaluated adhesive systems in uncured and cured conditions. Mean and standard deviation values of *in situ* DC (%) of the experimental groups are reported in Table 3. The adhesives Clearfil™ SE Bond containing 5% QAMP, Clearfil™ Protect Bond, and Clearfil™ SE Bond presented similar DC means (*p* > 0.05).

### 2.4. In Vitro Analysis of Degree of Conversion (DC)

*In vitro* DC (%) of Clearfil™ SE Bond containing 5% QAMP, Clearfil™ Protect Bond, and Clearfil™ SE Bond is indicated in Table 4. Considering ATR-FTIR data, the investigated self-etching adhesive systems demonstrated similar DC values (*p* > 0.05).

### 2.5. In Vitro Antibacterial Evaluation

The MIC and MBC values for the three bacterial strains are shown in Table 5. In all tests, the same endpoint was obtained for each of three replicates. Clearfil™ SE Bond containing 5% QAMP showed MIC values of 20 μL/mL for *S. mutans* and *A. naeslundii* and 10 μL/mL for *L. casei*. These results were lower than that verified for the Clearfil™ SE Bond, which presented MIC values of 80 μL/mL or higher. The positive control (Clearfil™ Protect Bond) was more effective than the experimental group only against *S. mutans*, demonstrating a MIC value of 10 μL/mL. For *L. casei* and *A. naeslundii*, Clearfil™ SE Bond containing 5% QAMP presented similar bacteriostatic effects to Clearfil™ Protect Bond.

Concerning the bactericidal effect, the three adhesive systems showed MBC values at the same concentration levels observed in MIC assay against *S. mutans* and *A. naeslundii*. Considering *L. casei*, MBC was higher than MIC for the experimental adhesive and controls. As previously observed for MIC, the bactericidal performance of Clearfil™ SE Bond containing 5% QAMP was also similar to the positive control against *L. casei* and *A. naeslundii* and higher than the negative control for all evaluated bacterial strains.

## 3. Discussion

Bond tests have been extensively used for investigating the performance of adhesive systems in order to improve strategies for increasing the durability of resin–dentin bonds [39]. The rationale is that a stronger adhesion between tooth and biomaterial can provide a better response to the resulting stress of resin polymerization and oral function. Several factors can affect the adhesive performance including heterogeneity of the dentin structure, changes on dentin surface after performing bur cutting and chemical treatments, inappropriate bond strategy, insufficient co-polymerization between adhesive and composite resin, *etc*. [39,40]. Therefore, it is imperative that new adhesives should be carefully evaluated regarding their bond properties before use in further clinical studies. Accordingly, this study reports that QAMP, a new antibacterial additive for self-etching adhesive systems, did not affect microtensile bond strength or the degree of conversion of a widely-used commercial adhesive. Thus the null-hypothesis was accepted.

In general, the experimental groups of adhesive systems demonstrated suitable μTBS values. However Clearfil™ Protect Bond showed a statistically significant decrease of μTBS after 12 months in water storage. The scientific literature reports that a long-lasting adhesive effect can be decreased by the release of agents from cured adhesive systems resulting in a low durability of restoration [16]. In a previous study, our research group demonstrated that Clearfil™ Protect Bond provided a rapid release of 47.2% of MDPB while Clearfil™ SE Bond containing QAMP showed a mean release of 5.1% of quaternary ammonium compounds after 30 days [27]. In addition, Clearfil™ Protect Bond contains a fluoride-containing adhesive that can also be released. Therefore the major release of chemical compounds from Clearfil™ Protect Bond such as MDPB and fluoride can be involved in the significant reduction of microtensile bond strength verified after the storage interval of 12 months.

Concerning the fracture mode, adhesive/mixed failures were mainly observed for the investigated adhesive systems and a low percentage of cohesive failure in dentin occurred only for Clearfil™ SE Bond containing 5% QAMP after 6 M and Clearfil™ Protect Bond after 6 and 12 months. According to the literature, this behavior is required for commercial adhesive systems when submitted to μTBS measurements since it indicates that the strength was applied to the resin–dentin interface and not to one of these substrates alone. In addition, these results indicate that the methods used for performing μTBS as cross-head speeds were suitably chosen.

Considering the micro-Raman spectroscopy analysis carried out for adhesive systems, the aromatic band (1609 cm^−1^) is typically used as an internal standard and no change is noticeable after polymerization [41]. On the other hand, changes in intensity of the aliphatic band (1639 cm^−1^) are due to a decrease in the number of remaining C=C double bonds during the polymerization process [40]. In particular, this usual behavior was observed for Clearfil™ SE Bond containing 5% QAMP in which no change in the *in situ* DC was verified. Additionally, Clearfil™ Protect Bond and Clearfil™ SE Bond showed DC values as previously reported [42,43]. These values were higher than 80% and can be related to the sample preparation that uses water for cutting procedure which can wash the unreacted monomer and can lead to increased results [42,43]. Considering that the same sample preparation was used for all groups, it is possible to conclude that the addition of QAMP to the commercial adhesive showed no influence on *in situ* DC, resulting in a suitable curing.

The *in vitro* approach carried out by ATR-FTIR also confirmed that QAMP did not significantly change the DC value of Clearfil™ SE Bond after its incorporation at a concentration of 5%. However, these results were remarkably lower than that obtained by the *in situ* procedure which can be strongly related to the particular *in vitro* conditions. Clearfil™ SE Bond results in an acid solution (pH near 2) and shows high water content *in vitro*, which can impair the polymerization reaction of the adhesive system [44]. On the other hand, the self-etching acidic primer can be buffered by the mineral content of dentin and enamel when applied to the tooth surface [43], which allows higher monomer conversion.

Innovative restorative materials with particular biological functions are not clinically useful if their original properties are effectively changed [18]. In this study, QAMP showed suitable response in a commercial adhesive system containing MDP, HEMA, and Bis-GMA as monomers in terms of μTBS and DC. Tests to assess DC over time after incorporating QAMP into self-etching adhesive systems should be performed in a future work.

Considering MIC/MBC results, Clearfil™ SE Bond containing 5% QAMP demonstrated similar bacteriostatic/bactericidal effects as the positive control (Clearfil™ Protect Bond) against *L. casei* and *A. naeslundii* which are pathogenic oral bacteria directly involved in caries and gingivitis, respectively. The positive control had better antibacterial effect than the experimental group only for *S. mutans*. This result can be related to the presence of fluoride in Clearfil™ Protect Bond, which might have provided an additional antibacterial effect. Moreover, QAMP was effective in conferring high antibacterial effects to Clearfil™ SE Bond by comparing the MIC/MBC results before and after its incorporation. Furthermore, an adhesive system containing QAMP demonstrated a lower release of quaternary compounds than the commercial MDPB-containing one [27], which can be considered an advantage in terms of prolonging the durability of the restoration and reducing toxicity to oral tissues. A further aspect to be highlighted is the antibacterial effect provided by the quaternary ammonium compounds fixed in the adhesives and the amount that was leached to the liquid medium. According to our experimental conditions, these two contributions were considered for MIC/MBC results. Further antimicrobial tests have to be performed in order to evaluate the influence of the leached compounds in the antimicrobial properties of these adhesive systems.

The clinical performance of new adhesive systems containing different chemical components must be investigated using the survival rates of restorations placed in non-carious cervical lesions [44,45]. Furthermore, discoloration and marginal integrity have to be evaluated since failure of restoration margin is a common reason for the replacement and repair of composite restorative [45]. Therefore clinical studies should be made in order to confirm these *in vitro* results after incorporating QAMP into adhesives.

## 4. Materials and Methods

### 4.1. Micro-Raman Spectroscopy of the Quaternary Ammonium Methacrylate Polymer (QAMP)

Micro-Raman spectroscopy was used for characterizing QAMP. Raman spectra of QAMP and raw materials (Eudragit™ E100 and *n-*octyl bromide) were collected at room temperature using a Bruker Raman Microscope Spectrometer (Bruker Optik GmbH, Ettlingen, Germany) with a 532 nm Nd:YAG laser line (20 mW) as an excitation source. Analysis were performed according to the following experimental parameters: charge-coupled device detector cooled at 208 K; spectral resolution of 3–5 cm^−1^; power on the sample of 2 mW; scanning range of 4000–200 cm^−1^; accumulation time of 20 s and scan number of 4. Opus spectroscopy software (version 6.5, Bruker Optik, Natick, MA, USA) was used to process the collected data.

### 4.2. Experimental Groups and Restorative Procedures

Three self-etching adhesive systems were evaluated: Clearfil™ SE Bond containing 5% QAMP, Clearfil™ Protect Bond (Kuraray Medical, Kurashiki, Japan) as positive control and Clearfil™ SE Bond (Kuraray Medical, Kurashiki, Japan) as negative control. Detailed composition and application mode of these commercial adhesive systems are described in Table 6.

The self-etching adhesive systems were applied onto the dentin in accordance with the manufacturer’s instructions. After light curing of each adhesive, three 1 mm-increments of composite resin Filtek™ Z350 XT (3M ESPE, St. Paul, MN, USA) were bonded to each dentin surface. Each increment was light cured with LED (LED Radii-cal, SDI, São Paulo, Brazil) for 30 s at 600 mW/cm^2^. All bond procedures were carried out by a single operator at room temperature and constant relative humidity.

### 4.3. Teeth Selection and Preparation

A total of forty-two extracted caries-free human third molars were used. Microtensile bond strength (μTBS) was performed using 30 teeth whereas analysis of *in situ* degree of conversion (DC) was carried out with 12 teeth. These teeth were collected after obtaining the patients’ informed consent under Protocol Number #17839/10 that was approved (#35/2011) by the Ethics Committee on Research Involving Human Subjects from the State University of Ponta Grossa, on 6 April 2011. The teeth were disinfected using 0.5% chloramine, stored in distilled water, and used within six months after extraction. A flat dentin surface was exposed after wet grinding the occlusal enamel on #180-grit silicon-carbide (SiC) paper. The exposed dentin surfaces were further polished on wet #600-grit SiC paper for 60 s to create a standardized smear layer.

### 4.4. Resin–Dentin Bond Strength (μTBS)

After storing the bonded teeth in distilled water for 24 h at 37 °C, thirty teeth (10 teeth per group) were longitudinally sectioned in both the mesio-distal and buccal-lingual directions across the bonded interface using a diamond saw in an Isomet 1000 machine (Buehler Ltd., Lake Bluff, IL, USA) at 300 rpm in order to obtain bonded sticks with a cross-sectional area of approximately 0.8 mm^2^. The number of premature debonded sticks per tooth during specimen preparation was recorded. The cross-sectional area of each stick was measured with a digital caliper (Absolute Digimatic, Mitutoyo, Tokyo, Japan). The bonded sticks of each tooth were divided to be tested immediately, after 6 and 12 months of storage in distilled water containing 0.4% sodium azide at 37 °C. The storage solution was not changed during μTBS experiments [46].

At each storage period, an individual bonded stick was attached to a Geraldelli’s device for μTBS using cyanoacrylate resin (Super Bonder Gel, Loctite, Piracicaba, Brazil). Each stick was stressed to failure using a universal testing machine (Kratos Dinamometros, São Paulo, Brazil) at a crosshead speed of 0.5 mm/min. Failure modes were evaluated in a light stereomicroscope (Nikon Eclipse E200, Melville, NY, USA) at 40× magnification and were classified as cohesive (failure exclusively within dentin or resin composite, C), adhesive (failure at resin/dentin interface, A), and adhesive/mixed (failure at resin/dentin interface that included cohesive failure of the neighboring substrates, A/M).

All tested sticks from the same tooth were averaged for statistical purposes. The prematurely debonded specimens were included in tooth mean. For each experimental group, μTBS mean was then obtained. Data were analyzed using two-way ANOVA (adhesive system and storage period) and Games Howell’s *post-hoc* test (α = 0.05) for pair-wise comparisons.

### 4.5. In Situ Analysis of Degree of Conversion (DC)

Twelve bonded third molars (4 teeth per group) were longitudinally sectioned in a mesio-to-distal direction across the bonded interface using a diamond saw in an Isomet 1000 machine at 300 rpm to prepare 2.0 mm resin-bonded dentin slices. Two slices from the center of each tooth were selected for the micro-Raman analysis.

The resin-bonded dentin slices (*n* = 8 for each experimental group) were individually and manually polished using SiC papers of decreasing abrasiveness (#1000, #1200, #1500, and #2000 grits). Specimens were ultrasonically cleaned for 10 min and then stored in water for 24 h at 37 °C before performing the analysis of DC.

Raman spectra were collected at room temperature using a computer-controlled laser Raman microscope spectrometer (Senterra, Bruker Optik, Ettlingen, Baden-Württemberg, Germany) with a 532 nm neodymium:yttrium-aluminium-garnet (Nd:YAG) laser line (20 mW) as an excitation source. Analyses were performed according to the following experimental parameters: charge-coupled device detector cooled at 208 K, spatial resolution of ≈3 μm, spectral resolution of ≈5 cm^−1^, power on the sample of 2 mW, scanning range of 1750–280 cm^−1^, accumulation time of 30 s and 6 co-additions, and 100× magnification (Olympus, London, UK) to a ≈1 μm beam diameter [35]. Spectra were taken in the middle of the hybrid layer, in an arbitrary area of the intertubular dentin. Two sites were examined per each dentin-adhesive slice.

Raman spectra of uncured adhesives were collected to identify the characteristic bands. Measurements were recorded three times with different specimens for each self-etching adhesive system. Opus spectroscopy software (version 6.5, Bruker Optik) was used to process the collected data. The double-bond content for converting monomer to polymer of each adhesive was calculated using the ratio between cured and uncured band intensities (Equation (1)):

(1) $$\%\;\text{conversion}=1-\{\frac{R\;(\text{cured})}{R\;(\text{uncured})}\}\times 100\%$$

where “*R*” is the ratio between aliphatic and aromatic band intensities at 1639 and 1609 cm^−1^ for cured and uncured adhesives.

Considering that the obtained values were normally distributed (Kolmogorov–Smirnof test), DC (%) was evaluated using a one-way ANOVA and Tukey’s *post-hoc* test (α = 0.05).

### 4.6. In Vitro Analysis of Degree of Conversion (DC)

The *in vitro* DC was investigated by Fourier transform infrared spectroscopy (FTIR) with an attenuated total reflectance (ATR) device (Spectrum 100, PerkinElmer, Shelton, CT, USA) at 24 °C, under 64% relative humidity. The self-etching adhesive systems evaluated were: Clearfil™ SE Bond containing 5% QAMP, Clearfil™ Protect Bond and Clearfil™ SE Bond (negative control). In order to obtain DC, three samples of each uncured adhesive system and five thin films (0.2–0.3 mm thick) of each cured adhesive were applied on the plate with ZnSe crystal for acquiring the absorption spectra.

The absorption spectra of cured and uncured adhesive systems were collected from 4000 to 650 cm^−1^ with 32 scans at 4 cm^−1^. The ratio between band intensities of C=C aliphatic methacrylate group (1639 cm^−1^) and the reference C···C aromatic ring (1609 cm^−1^) was obtained for cured and uncured adhesives. The DC (%) was than calculated using Equation 1. Data were analyzed by one-way ANOVA and Tukey’s *post-hoc* test (α = 0.05). The SPSS Statistics software, version 20.0 (SPSS Statistics, IBM Corporation, Somers, NY, USA) was used to perform all the statistical analysis in this study.

### 4.7. In Vitro Antibacterial Evaluation

The antibacterial properties of Clearfil™ SE Bond containing 5% QAMP and controls were investigated by *in vitro* susceptibility tests of minimum inhibitory/minimum bactericidal concentrations as previously reported [25,27,47].

In brief, plates containing Mueller-Hinton (MH) agar with 5% sheep blood were inoculated with strains of *Streptococcus mutans* (ATCC 25175), *Lactobacillus casei* (ATCC 393), and *Actinomyces naeslundii* (ATCC 12104) from previously standardized inocula at a concentration of 5 × 10^5^ CFU/mL that achieved the turbidity of 0.5 at McFarland scale (1.5 × 10^8^ CFU/mL) by swab streaking [39,40]. Volumes of 10, 20, 40, and 80 μL of Clearfil™ SE Bond containing 5% QAMP, obtained by using equal aliquots of primer and bond, positive and negative controls were spread onto the bottom of hemolysis tubes, air-thinned and light-cured with light-emitting diode (LED) for 20 s [17]. The final volume and exposed surface area were not controlled during the *in vitro* antibacterial experiments. Afterwards, 1 mL of brain-heart infusion (BHI) broth containing cultures of each microorganism at a concentration of 5 × 10^5^ CFU/mL was added. These tubes were shaken once after addition of BHI broth and cultured under microaerophilic conditions (5% O_2_, 10% CO_2_, 85% N_2_) during 24 h at 35 ± 0.5 °C [48]. The bacterial growth was estimated by changes in optical density (OD) considering the measurements taken before and after incubation. For OD evaluation, 200 μL of each culture medium was transferred to a 96-well microplate and the absorbance was measured at 450 nm using a spectrophotometric plate reader (EL800, Bio-tek Instruments, Winooski, VT, USA). Tests were performed in triplicate with fresh BHI serving as blank control. The minimum inhibitory concentration (MIC) was defined as the endpoint where no turbidity might be detected with respect to the controls.

In order to determine the minimum bactericidal concentration (MBC), subcultures were obtained by spreading a 100 μL-aliquot from each test tube without turbidity on Mueller-Hinton agar plates supplemented with 5% sheep blood. Plates were incubated under microaerophilic conditions for 24 h at 35 ± 0.5 °C. The MBC value was determined as the lowest concentration of each adhesive system that provided no bacterial growth.

## 5. Conclusions

In summary, the experimental results demonstrated that QAMP did not change the required adhesive properties of resin–dentin bond strength and degree of conversion and further provided antibacterial effects for the evaluated adhesive system against usual pathogenic oral bacteria. These data support a promising potential for the use of quaternary ammonium methacrylate polymers in dental adhesive to promote the additional advantages of preventing caries lesions and prolonging the durability of dental restorations.

## Figures and Tables

**Table 1.** Mean and standard deviation values of overall resin-dentin bond strength (MPa) from the evaluated adhesive systems.

Adhesive system	Storage period		

	Immediately	6 months	12 months
Clearfil™ SE Bond containing 5% QAMP	34.88 ± 8.71 Aa	33.43 ± 5.47 Aa	32.50 ± 5.94 Aa
Clearfil™ Protect Bond	37.23 ± 6.71 Aa	39.44 ± 3.25 Aa	28.20 ± 8.09 Ba
Clearfil™ SE Bond	34.69 ± 5.95 Aa	32.08 ± 10.97 Aa	31.27 ± 9.30 Aa

Groups identified with the same letters are not significantly different (Tukey’s test, *p* > 0.05), with uppercase letters (for each adhesive system) in rows or lowercase letters (for each storage period) in columns.

**Table 2.** Fracture mode and premature debonded specimens from the evaluated adhesive systems (*).

Adhesive system	Storage period											

	Immediately				6 months				12 months			
		
	A/M	C	Debond	T	A/M	C	Debond	T	A/M	C	Debond	T
Clearfil™ SE Bond containing 5% QAMP	36(80)	0(0)	9(20)	45	37(92.5)	2(5)	1(2.5)	40	34(94.5)	0(0)	2(5.5)	36
Clearfil™ Protect Bond	35(79.6)	0(0)	9(20.4)	44	35(83.3)	1(2.4)	6(14.3)	42	34(91.9)	1(2.5)	3(8.1)	37
Clearfil™ SE Bond	35(71.4)	0(0)	14(28.6)	49	35(81.4)	0(0)	8(18.6)	43	35(83.3)	0(0)	7(16.7)	42

* Results are shown as number, percentage of specimens and total of sticks;

Legend: A/M, adhesive/mixed fracture mode; C, cohesive fracture mode; Debond, premature debonded specimens; T, total.

**Table 3.** Mean and standard deviation values of *in situ* analysis of degree of conversion (%) for adhesive systems.

Adhesive systems		
Clearfil™ SE Bond containing 5% QAMP	Clearfil™ Protect Bond	Clearfil™ SE Bond
89 ± 6 ^a^	85 ± 11 ^a^	88 ± 3 ^a^

Groups identified with the same lowercase letters are not significantly different (Tukey’s test, *p* > 0.05).

**Table 4.** Mean and standard deviation values of *in vitro* analysis of degree of conversion (%) for adhesive systems.

Adhesive systems		
Clearfil™ SE Bond containing 5% QAMP	Clearfil™ Protect Bond	Clearfil™ SE Bond
39.7 ± 2.9 ^a^	39.8 ± 4.9 ^a^	40.0 ± 3.0 ^a^

Same lowercase letters indicate (*p* > 0.05) in columns.

**Table 5.** Minimum inhibitory concentration (MIC) and minimum bactericidal concentration (MBC) values for each adhesive system against three pathogenic oral bacteria.

Adhesive system	MIC/MBC values (μL/mL)		

	*Streptococcus mutans* (ATCC 25175)	*Lactobacillus casei* (ATCC 393)	*Actinomyces naesludii* (ATCC 12104)
Clearfil™ SE Bond containing 5% QAMP	20/20	10/20	20/20
Clearfil™ Protect Bond	10/10	10/20	20/20
Clearfil™ SE Bond	80/80	80/>80	80/80

**Table 6.** Adhesive systems: composition (batch number) and application mode.

Adhesive systems	Composition (batch number)	Application mode
Clearfil™ SE Bond containing 5% QAMP	Primer: MDP, HEMA, hydrophilic dimethacrylate, *di*-camphorquinone, *N*,*N*-diethanol-*p*-toluidine, water, QAMP. (00954A) Bond: MDP, Bis-GMA, HEMA, hydrophobic dimethacrylate, *di*-camphorquinone, *N*,*N*-diethanol-*p*-toluidine, silanated colloidal silica. (01415A)	Application of two coats of the primer under pressure (20 s); Gentle air stream (10 s at 20 cm) after application of each coat; Application of one coat of the adhesive (15 s); Gentle air stream to make the bond film uniform (3 s at 20 cm); Light cured (10 s at 600 mW/cm^2^).
Clearfil™ Protect Bond	Primer: MDPB, MDP, HEMA, hydrophilic dimethacrylate, water. (00081B) Bond: MDP, HEMA, Bis-GMA, hydrophobic dimethacrylate, *di*-camphorquinone, *N*,*N*-diethanol-*p*-toluidine, silanated colloidal silica, surface-treated sodium fluoride. (00134B)	
Clearfil™ SE Bond	Primer: MDP, HEMA, hydrophilic dimethacrylate, *di*-camphorquinone, *N*,*N*-diethanol-*p*-toluidine, water. (00954A) Bond: MDP, Bis-GMA, HEMA, hydrophobic dimethacrylate, *di*-camphorquinone, *N*,*N*-diethanol-*p*-toluidine, silanated colloidal silica. (01415A)	

Abbreviations: MDP, 10-methacryloxydecyl dihydrogen phosphate; HEMA, 2-hydroxyethyl methacrylate; Bis-GMA, bisphenol-glycidyl methacrylate; MDPB, 12-methacryloyloxydodecyl pyridinium bromide; and QAMP, [poly(dimethylaminoethyl methacrylate-*co*-octyldimethylammonium ethyl methacrylate bromide-*co*-methyl methacrylate-*co*-butyl methacrylate)].
